# Policy instruments for the governance of the social drivers of health data in clinical and research settings: a policy mapping brief

**DOI:** 10.3389/fpubh.2024.1369790

**Published:** 2024-11-14

**Authors:** Yulia A. Levites Strekalova, Xiangren Wang, Sara Midence, Alexander Quarshie

**Affiliations:** ^1^Department of Health Services Research, Management, and Policy, College of Public Health and Health Professions, University of Florida, Gainesville, FL, United States; ^2^Clinical and Translational Science Institute, University of Florida, Gainesville, FL, United States; ^3^Morehouse School of Medicine, Atlanta, GA, United States

**Keywords:** social drivers of health, Z codes, PhenX toolkit, policy options, health equity

## Abstract

This paper maps policy instrument use for the social drivers of health (SDoH) data governance in clinical and research settings. In the United States, Centers for Medicare and Medicaid Services (CMS) and National Institutes of Health (NIH) advocate for standardized data capture. Yet, challenges persist, including limited adoption of CMS-issued SDoH risk codes and gaps in reporting SDoH in clinical trial literature. The mapping across clinical and research SDoH reporting emerges as a comprehensive solution that requires policy support. Specifically, the findings presented in this paper support future policy development through regulatory instruments, fiscal incentives, and knowledge exchange. Actionable recommendations for the United States and international contexts include convening interdisciplinary taskforces, developing agency guidelines for process evaluation, and establishing ethical principles for SDoH data use.

## Introduction

The role of social drivers of health (SDoH) has been extensively documented and linked to health outcomes (1, 2). There is a wide-reaching consensus on the importance of capturing the SDoH systematically and rigorously, and research increasingly demonstrates how SDoH contribute to disparities observed across various demographic groups (3–6). Capturing SDoH data in clinical practice is essential for addressing the complex interplay between social factors and health outcomes. Similarly, capturing SDOH in clinical research is essential for understanding and addressing the multifaceted factors that influence patient outcomes. However, the unstructured format of SDoH data in electronic health records limits the ability to address these issues effectively (7). Despite the availability of SDoH data in national sources, there is inconsistency in data collection within health systems, indicating an opportunity for improvement in capturing comprehensive SDoH data. High noncompletion rates in capturing SDoH data underscore the need for evidence-based guidelines to improve data collection in underserved populations (7). Furthermore, barriers such as incentives, training, privacy, and ethical use of data need to be addressed to fully integrate social risk assessment into healthcare delivery systems and translational research (8, 9). Therefore, there is a need for focused efforts to identify and implement evidence-based policy instruments that support the SDoH data governance in both clinical and research contexts.

Health data governance requires established guidelines and procedures to ensure the availability, integrity, security, and usability of both structured and unstructured data and facilitate its strategic management to enhance organizational effectiveness and quality of care (10). The need for SDoH data governance spans clinical practice and clinical research domains as the research-to-practice and practice-to-research issues in SDoH data capture and sharing are multifaceted, involving organizational, ethical, technical, and system behavior challenges (11–13). SDoH data are frequently fragmented across multiple health data sources and healthcare service deliveries (14–16). Healthcare and social service providers can communicate data at the time of care if they have access to interoperable data on particular social hazards and corresponding treatments (13). Population health management, documentation of social needs, intervention implementation and evaluation, and model refinement of care delivery may all be enhanced at the practice level with aggregated social data (4, 17–19). Furthermore, real-world data on SDoH are often derived from multiple sources that include electronic health records, medical claims, and patient-generated data (17, 20–22). While there is a growing recognition of the importance of data sharing to strengthen academic research and clinical practices, it comes with calls for policies to improve data management planning, harmonization of practices, and ethical sourcing and use data (14, 23, 24).

Health policy instruments can be categorized into three main types, each serving distinct functions in governance and public health (25, 26). Legislative and regulatory instruments involve laws, regulations, and standards that mandate or restrict certain behaviors to enforce compliance and protect public health. Economic and fiscal instruments include taxes, subsidies, and pricing strategies designed to influence individual and organizational behaviors. Cooperative and knowledge translation instruments rely on partnerships, coordination, and voluntary agreements between stakeholders, including public-private partnerships and community collaborations, to achieve health objectives without direct regulation. Lastly, we also propose and articulate knowledge translation as a policy instrument that involves the collection and dissemination of scientific evidence to inform policy decisions and promote evidence-based practices, ensuring that health policies are grounded in the latest and most reliable knowledge. Together, these instruments stand to create a comprehensive policy mix that addresses the complexities of SDoH data governance, ensuring that data are collected, managed, and utilized effectively to improve public health outcomes.

In this policy brief, we aim to (1) discuss the existing policy efforts made to develop and collect SDoH and (2) suggest future policy development and implementation efforts that can help and direct the SDoH data governance, namely, the collection and use of SDoH data in clinical practice, quality improvement, and partnerships between research and practice.

## Analysis of policy instruments and implications for SDoH data governance

Policy can support and provide a framework for SDoH data capture in healthcare and research contexts. In the United States, the Centers for Medicare and Medicaid Services (CMS) produced guidelines and created pathways to develop the SDoH data capture systems (1, 27–29). However, because of the complexity of the SDoH domain, there is no consensus on the approach to the SDoH screening and the set of common SDoH elements. As a governmental agency, the CMS actively uses policy to regulate, promote, and encourage this capture (30). Similarly, the National Institute of Health (NIH) and other funding agencies are employing policy tools to promote, encourage, and ensure the standardized capture of the SDoH for research (28, 31). Strategic use of the policy instruments rooted in ethical data governance can promote the systematic, standardized capture of social drivers of health, and foster research-practice partnerships. Next, we will discuss the existing policy efforts for the SDoH data governance in clinical and research domains.

### Regulatory policies

Regulatory policy instruments can provide the guidelines and expectations of required and recommended screening for and capturing of the SDoH. Within patient care, the CMS has, for many years, encouraged the use of Z codes and now requires their inclusion in specific reports. In the United States, the CMS has issued detailed guidelines for systematically implementing Z codes to report SDoH in clinical environments (3). The assessment requires the use of tested and validated tools that cover essential SDoH domains like housing instability, food insecurity, and utility difficulties. Currently, only a minority of patients are screened for SDoH, but the introduction of required SDoH screening for hospital admissions is likely to increase the proportion of the United States population that receives screening (32, 33). Within the context of clinical research in the United States, the NIH requires that human subject studies report the demographics of research participants for planned and actual enrollment. This is another example of a regulatory policy instrument that mandates SDoH data capture (28). While only basic demographics are currently required to be captured for federally funded research studies, NIH efforts are underway to develop a new set of minimum common data elements, including the SDoH (34).

### Fiscal policies

The implementation of SDoH screening requires a technical and financial infrastructure that accounts for the data collection burden. Although the CMS has dedicated considerable resources to advancing the use of Z codes, their widespread integration into healthcare documentation practices still needs to be improved (6). Recent findings reveal that a mere 5% of Medicare recipients have SDoH-related Z codes recorded in the EMR (7, 8). This number is thought to underrepresent the true prevalence of SDoH influences on the health of Medicare beneficiaries, suggesting a discrepancy between recorded data and the actual prevalence of SDoH within the United States population. The CMS is moving toward using economic instruments to promote SDoH screening policies through the proposed reimbursement strategies for SDoH screening, which will reward practices for the time spent conducting SDoH assessments. In 2024, the CMS implemented a new GXXX5 code for administering a standardized, evidence-based SDoH risk to enhance the understanding of a patient’s social history and its influence on health emphasizing the necessity of follow-up actions and interventions after the assessment. Further support for SDoH data capture can be created through fiscal policy instruments such as financial subsidies, grants, and incentives that support the development of legal, technical, and operational infrastructure for data capture, harmonization, management, storage, and use (24).

### Cooperative policies

While regulatory and economic policies can be effective, they are also limited in what they can achieve if implemented in silos. Cooperative policies are the third policy instrument actively applied by government agencies to promote knowledge exchange and coordination of actions. The most common implementation of collaborative policy instruments involves the establishment of taskforces that develop recommendations and pathways for inter-organizational collaboration. As a policy instrument, establishing a taskforce requires a financial and staffing commitment for interagency partnerships among clinical, research, industry, community, and governmental organizations. Ultimately, all agencies are interested in the same goal: identifying social drivers and finding strategies to address them. However, research and practice are at risk of uncoordinated information flow and efforts to establish common data capture approaches (35).

In response to the need for standardized SDoH data collection, the National Institute on Minority Health and Health Disparities (NIMHD) led a task force effort to develop a set of consensus-based instruments for use in research studies to assess SDoH and standardize the SDoH screening protocols for clinical research (31). The instruments were selected and evaluated for inclusion in the “Consensus Measures for Phenotypes and eXposures” PhenX toolkit (36). With the introduction of the PhenX Toolkit in May 2020, data capture protocols were encapsulated, offering assessments at the individual and structural levels. Developed protocols included a Core SDoH collection with 16 measurements recommended for all research studies. The Core collection includes demographics (e.g., ethnicity and race, age, gender identity, annual family income, and employment status) and social driver variables that address structural and individual SDoH. Active awards from the NIH National Institute for Minority Health and Health Disparities promote the use of PhenX SDoH Core toolkits. For example, the Research Centers in Minority Institutions (RCMI) program uses PhenX as a guide to develop common data models and protocols for SDoH capture (45). Through the establishment of the RCMI Clinical Research Network for Health Equity (CRNHE), several dynamic clinical networks will collaborate to reach a unanimous agreement on the PhenX SDoH core toolkits. Consensus metrics for SDoH in clinical research, developed by the PhenX Toolkit, are an essential first step toward social driver assessment standards (31). The toolkit supports a transparent approach to include SDoH considerations in clinical research (37).

### Knowledge translation policies

Knowledge translation as a policy instrument for SDoH data governance leverages practice-based research to bridge the gap between data production and policy implementation, ensure that data capture and use are both informed and actionable (38–40), and integrate evidence-based strategies and stakeholder engagement into the policy-making process (41). Knowledge translation systematically moves research evidence into practice and policy. This process can be guided by the theory of change frameworks that emphasize the iterative of policy development, ensuring that evidence is not only scientifically plausible but also acceptable and practical for implementation (42, 43). As discussed earlier in this brief, there are significant areas of overlap in SDoH data use for clinical and research purposes, which can be further supported through knowledge transfer practices and accepted cross-referencing of the SDoH data elements.

With the increasing reliance on real-world data, the significance of integrating Z codes, PhenX protocols, and SDoH screening tools has become more pronounced. Partnerships between research and practice can be bolstered by employing harmonized coding systems in tandem with validated SDoH screening instruments. Numerous instruments have been developed to collect data (13, 14), and collecting comprehensive SDoH data requires time and a level of rapport and trust that can be difficult to establish in brief clinical encounters (44). The limitations of collecting SDoH variables thus become a significant barrier, impeding the ability of healthcare providers to fully understand and address the broader social and economic factors that influence their patients’ health (26). Integrating research-focused PhenX data and clinically focused Z codes can be achieved by explicitly mapping Z codes onto research instruments and the gradual rollout of requirements to screen for the social drivers of research participants’ health.

When considered broadly, most of the SDoH domains represented by PhenX and can be mapped to Z codes as shown in Table 1. However, mapping the Z codes and PhenX toolkit reveals several disconnects. Notable exceptions from the Z codes are demographic data, such as race, ethnicity, age, and current address, which are expected to be captured at patient intake. While this is a reasonable expectation, demographic point-of-care data capture varies among clinical practices. It may omit questions about birthplace and differentiation between sex assigned at birth and self-reported gender (21). Other constructs related to social integration (Z60), housing stability (Z59), safety (Z62), domestic violence (Z63), and material security (Z58) are included in the Z codes but not in the PhenX Core instruments. This finding highlights the primary focus on interpersonal relationships and factors related to family (27). When assessed in clinical care, these Z codes capture various social issues influencing the patient’s health and well-being. Finally, access to health services identified in PhenX map to a Z code (Z75) outside the Z55-65 range that identifies health hazards related to socioeconomic and psychosocial circumstances.

**Table 1:** SDoH domain mapping.

Z Codes (Z55-Z65)	PhenX core instruments
Z55: Problems related to education and literacy	Educational Attainment Individual
Z55: Problems related to education and literacy	English Proficiency
Z55: Problems related to education and literacy	Health Literacy
Z56: Problems related to employment and unemployment	Current Employment Status
Z57: Occupational exposure to risk factors	Occupational Prestige
Z58: Problems related to physical environment	N/A
Z59: Problems related to housing and economic circumstances	Annual Family Income
Z59: Problems related to housing and economic circumstances	Food Insecurity
Z59: Problems related to housing and economic circumstances	N/A
Z59: Problems related to housing and economic circumstances	Health Insurance Coverage
Z60: Problems related to social environment	N/A
Z61: Loss of love relationship in childhood	N/A
Z62: Problems related to upbringing	N/A
Z63: Other problems related to a primary support group, including family circumstances	N/A
Z64: Problems related to certain psychosocial circumstances	N/A
Z65: Problems related to other psychosocial circumstances	N/A
Z65: Problems related to other psychosocial circumstances	Sexual Orientation
[Z75: Problems related to medical facilities and other health care]	Access to Health Services
N/A	Birthplace
N/A	Current Address
N/A	Current Age
N/A	Ethnicity and Race
N/A	Sex Assigned at Birth

## Findings and actionable recommendations

The policy mapping presented above focuses on the United States policy context. However, the selection of policy instruments can inform policy efforts in other national and regional applications. To enhance the capture and use of SDoH data within clinical and research settings, we provide the following actionable recommendations: a strategic direction and a detailed plan of action, each supported by a brief explanation drawing from current evidence and best practices (6).

### Convene an interdisciplinary task force of health policy experts, clinicians, data analysts, and patient advocates to oversee the collection and integration of SDoH data into health and research systems

It would be essential to convene a team that represents a diversity of perspectives on SDoH data governance and includes the representation of community and patient representatives (41). The task force should first identify barriers such as inconsistent definitions and data quality issues and consider the legal and organizational shortcomings in health data systems. The task force should advocate for resources to enhance data linkage and text classification methods, which show promise for improving SDoH data integration. By addressing these aspects, the task force can effectively advocate for the necessary resources and policies to integrate SDoH data into health systems, ultimately improving health outcomes and equity.

### Develop agency guidelines for cross-mapping Z codes and PhenX toolkits with input from open comments

By collecting stakeholders’ suggestions through available comments, the agency ensures that the guidelines are developed with transparency and inclusivity. The stakeholders will be able to contribute to ensuring that the policies are well-rounded and consider the needs (30). To develop agency guidelines for cross-mapping Z codes and PhenX toolkits with input from open comments, it is essential to adopt a multi-stakeholder approach that ensures transparency and inclusivity. Additionally, the guidelines can incorporate ethical considerations surrounding the use of real-world and open-source neighborhood data.

### Provide supplements for process evaluation for SDoH data capture at the point of care

Offer additional funding or resources to support the assessment of SDoH data capture processes. This includes assessing workflows, data accuracy and conciseness, and the impact on patient outcomes (9). By understanding these processes, healthcare and research teams can refine data capture methods to be more effective and less intrusive.

### Establish guiding principles and requirements for clinical and research SDoH data capture and use

Define a set of guiding principles and requirements for the capture and use of SDoH data in clinical and research contexts. These principles should emphasize ethical considerations (consent and data sovereignty), data quality, privacy, and the meaningful use of data in improving health outcomes. Furthermore, the Findable, Accessible, Interoperable, and Reusable (FAIR) principles should be adopted to promote rigorous and reproducible data collection and management practices.

### Encourage the consistent and ethical use of SDoH data

Advocate for policies that promote the consistent and ethical use of SDoH data, ensuring that it is used to improve patient care and health outcomes without compromising patient privacy or autonomy (14).

## Conclusion

The development of robust policy instruments for the governance of SDoH data is critical to advancing health equity through the systematic capture and use of data in both clinical and research contexts. Regulatory instruments, such as those issued by the U.S. Centers for Medicare and Medicaid Services, establish essential frameworks for standardized SDoH data collection. Yet, gaps remain in compliance and data uniformity, necessitating further refinement of these policies to align with evidence-based practices. Economic and fiscal policies, including incentives for SDoH screening, must be expanded to address the financial and infrastructural burdens of data collection, ensuring that healthcare providers are adequately supported. Cooperative instruments, exemplified by the task force-led development of the PhenX SDoH toolkit, underscore the importance of cross-sector collaboration in harmonizing data collection practices across research and clinical settings. Furthermore, the integration of knowledge translation instruments will enable the alignment of real-world clinical data with research outcomes through tools such as ICD-10 and PhenX. Ethical considerations surrounding privacy and data sovereignty must be embedded within governance frameworks to safeguard patient autonomy while enabling the actionable use of SDoH data. Lastly, interdisciplinary task forces should be institutionalized to provide ongoing guidance and ensure the adaptive refinement of SDoH data governance practices, with continuous process evaluations that inform policy adjustments and enhance the efficacy of data-driven interventions aimed at addressing health disparities.

Data governance challenges create barriers in SDoH data sharing, integration, and utilization in clinical practice and research. Strategic use of regulatory, fiscal, cooperation, and knowledge transfer policy instruments is essential for informed public health decision-making and continued progress toward health equity. As the healthcare landscape continues to evolve, the need for such integrative and inclusive approaches in clinical practice and research become increasingly vital. By articulating the types of policy instruments for SDoH data governance, this paper aims to spark further discussion and participatory action toward a more inclusive and effective healthcare system that can better recognize the complex interplay of social, economic, and environmental factors in health and health disparity. This paper identifies existing gaps in the adoption of CMS-issued Z codes and SDoH reporting in clinical trials and offers a structured pathway to bridge these gaps through targeted policy development and application. The actionable recommendations discussed above are grounded in established practices and evidence. Specifically, by proposing concrete actions such as financial incentives and regulatory mandates, this paper emphasizes the importance of policy instruments in facilitating comprehensive data capture. Importantly, the inclusion of ethical principles ensures that policies uphold patient agency, privacy, and consent, thereby promoting equitable health outcomes.

In conclusion, this paper presents a roadmap for policymakers and healthcare leaders to transform SDoH data governance through structured and evidence-based policy tools, advancing the capacity of healthcare systems to address the underlying social, economic, and environmental drivers of health disparities. While addressing specific U.S. policy contexts, this policy mapping brief also provides a framework for future cross-national comparative studies.
